# Supplementary material for: Hot spot prediction in protein-protein interactions by an ensemble system
Source: BMC Syst Biol. 2018 Dec 31;12(Suppl 9):132. doi: 10.1186/s12918-018-0665-8 (PMC6311905; doi:10.1186/s12918-018-0665-8)
Supplement: Supplementary file 2 — All relevant MATLAB code. A simple Matlab implement of our predictor. (ZIP 19,742 kb) [file 12918_2018_665_MOESM2_ESM.zip › Additional file 2/Read ASA/ASA website/dbMPIKT/2KSOA.html]

 NetSurfP - Protein Surface Accessibility and Secondary Structure Predictions

  

|  |  |  |
| --- | --- | --- |
|  |  | NetSurfP - Protein Surface Accessibility and Secondary Structure Predictions  Technical University of Denmark |

  


---

```
# For publication of results, please cite:
# A generic method for assignment of reliability scores applied to solvent accessibility predictions.
# Bent Petersen, Thomas Nordahl Petersen, Pernille Andersen, Morten Nielsen and Claus Lundegaard
# BMC Structural Biology 2009, 9:51 doi:10.1186/1472-6807-9-51
#
# Column 1: Class assignment - B for buried or E for Exposed - Threshold: 25% exposure, but not based on RSA
# Column 2: Amino acid
# Column 3: Sequence name
# Column 4: Amino acid number
# Column 5: Relative Surface Accessibility - RSA
# Column 6: Absolute Surface Accessibility
# Column 7: Z-fit score for RSA prediction
# Column 8: Probability for Alpha-Helix
# Column 9: Probability for Beta-strand
# Column 10: Probability for Coil
E H  2KSOA                  1    0.936 170.313   0.187   0.003   0.003   0.994
E H  2KSOA                  2    0.633 115.088  -1.368   0.018   0.019   0.964
E H  2KSOA                  3    0.597 108.649  -0.814   0.018   0.047   0.935
E H  2KSOA                  4    0.560 101.846  -0.239   0.018   0.088   0.893
E H  2KSOA                  5    0.441  80.218  -1.023   0.018   0.088   0.893
E H  2KSOA                  6    0.520  94.606  -0.974   0.018   0.088   0.893
E S  2KSOA                  7    0.366  42.942  -1.063   0.018   0.047   0.935
E S  2KSOA                  8    0.427  50.044  -1.852   0.018   0.019   0.964
E G  2KSOA                  9    0.627  49.321  -1.761   0.018   0.019   0.964
E L  2KSOA                 10    0.502  91.861  -1.277   0.018   0.019   0.964
B V  2KSOA                 11    0.218  33.537  -1.209   0.018   0.019   0.964
E P  2KSOA                 12    0.662  93.895  -2.453   0.016   0.005   0.979
E R  2KSOA                 13    0.613 140.285  -2.361   0.003   0.003   0.994
B G  2KSOA                 14    0.251  19.785  -1.015   0.003   0.003   0.994
E S  2KSOA                 15    0.434  50.853  -0.303   0.005   0.015   0.979
B H  2KSOA                 16    0.260  47.276  -1.101   0.181   0.016   0.803
E M  2KSOA                 17    0.566 113.257  -0.818   0.339   0.016   0.645
E T  2KSOA                 18    0.320  44.315   1.275   0.522   0.016   0.462
B V  2KSOA                 19    0.024   3.673   0.809   0.970   0.001   0.030
B S  2KSOA                 20    0.262  30.765   0.789   0.970   0.001   0.030
E E  2KSOA                 21    0.443  77.410   1.565   0.970   0.001   0.030
B W  2KSOA                 22    0.053  12.867   1.081   0.970   0.001   0.030
B L  2KSOA                 23    0.051   9.265   0.075   0.923   0.002   0.076
E E  2KSOA                 24    0.596 104.069   1.226   0.858   0.002   0.139
E S  2KSOA                 25    0.420  49.236   0.536   0.694   0.003   0.303
B I  2KSOA                 26    0.112  20.702   0.954   0.246   0.004   0.750
E K  2KSOA                 27    0.562 115.521   0.757   0.053   0.005   0.942
B M  2KSOA                 28    0.110  22.051   0.403   0.321   0.003   0.675
E Q  2KSOA                 29    0.569 101.588   0.742   0.622   0.015   0.363
E Q  2KSOA                 30    0.485  86.621   1.511   0.717   0.014   0.269
B Y  2KSOA                 31    0.082  17.545   0.621   0.802   0.014   0.185
B T  2KSOA                 32    0.266  36.866   1.467   0.879   0.010   0.111
E E  2KSOA                 33    0.712 124.299   1.289   0.923   0.002   0.076
E H  2KSOA                 34    0.282  51.259   2.023   0.970   0.001   0.030
B F  2KSOA                 35    0.037   7.386  -0.462   0.923   0.002   0.076
E M  2KSOA                 36    0.446  89.185   1.135   0.923   0.002   0.076
E A  2KSOA                 37    0.732  80.633   1.215   0.858   0.002   0.139
E A  2KSOA                 38    0.376  41.424   0.846   0.321   0.003   0.675
E G  2KSOA                 39    0.564  44.371  -0.413   0.053   0.005   0.942
B Y  2KSOA                 40    0.077  16.348   0.738   0.018   0.019   0.964
E T  2KSOA                 41    0.441  61.153   0.129   0.018   0.019   0.964
B A  2KSOA                 42    0.253  27.870   0.107   0.113   0.043   0.844
B I  2KSOA                 43    0.120  22.274   0.530   0.802   0.014   0.185
E E  2KSOA                 44    0.566  98.810   0.869   0.802   0.014   0.185
E K  2KSOA                 45    0.269  55.436   1.419   0.802   0.014   0.185
B V  2KSOA                 46    0.019   2.997   1.162   0.717   0.014   0.269
E V  2KSOA                 47    0.312  48.031   1.162   0.522   0.016   0.462
E Q  2KSOA                 48    0.583 104.070   1.553   0.257   0.016   0.727
B M  2KSOA                 49    0.060  12.046   0.983   0.115   0.016   0.868
E T  2KSOA                 50    0.403  55.910   1.497   0.018   0.019   0.964
E N  2KSOA                 51    0.519  75.996   1.253   0.858   0.002   0.139
E D  2KSOA                 52    0.667  96.144   1.874   0.923   0.002   0.076
B D  2KSOA                 53    0.137  19.785   0.928   0.923   0.002   0.076
B I  2KSOA                 54    0.036   6.623  -0.179   0.923   0.002   0.076
E K  2KSOA                 55    0.538 110.728   1.795   0.923   0.002   0.076
E R  2KSOA                 56    0.658 150.796   1.797   0.782   0.003   0.216
B I  2KSOA                 57    0.108  20.017   1.104   0.406   0.004   0.590
E G  2KSOA                 58    0.503  39.555  -0.149   0.109   0.005   0.886
B V  2KSOA                 59    0.084  12.926   0.020   0.018   0.019   0.964
E R  2KSOA                 60    0.499 114.271   0.745   0.018   0.019   0.964
E L  2KSOA                 61    0.459  84.080   0.466   0.018   0.019   0.964
E P  2KSOA                 62    0.377  53.425  -0.484   0.622   0.015   0.363
E G  2KSOA                 63    0.364  28.615  -0.763   0.802   0.014   0.185
B H  2KSOA                 64    0.146  26.612  -0.035   0.879   0.010   0.111
B Q  2KSOA                 65    0.129  23.057   0.265   0.970   0.001   0.030
E K  2KSOA                 66    0.385  79.174   1.035   0.970   0.001   0.030
E R  2KSOA                 67    0.315  72.066   2.263   0.970   0.001   0.030
B I  2KSOA                 68    0.021   3.867   1.159   0.970   0.001   0.030
B A  2KSOA                 69    0.106  11.637   0.516   0.988   0.000   0.012
E Y  2KSOA                 70    0.450  96.272   1.589   0.970   0.001   0.030
B S  2KSOA                 71    0.071   8.345   0.328   0.970   0.001   0.030
B L  2KSOA                 72    0.021   3.882   0.885   0.970   0.001   0.030
E L  2KSOA                 73    0.439  80.308   1.841   0.970   0.001   0.030
E G  2KSOA                 74    0.410  32.275   2.069   0.970   0.001   0.030
B L  2KSOA                 75    0.041   7.434   0.295   0.970   0.001   0.030
E K  2KSOA                 76    0.304  62.636   1.317   0.970   0.001   0.030
E D  2KSOA                 77    0.610  87.872   1.283   0.923   0.002   0.076
E Q  2KSOA                 78    0.406  72.529   0.724   0.923   0.002   0.076
B V  2KSOA                 79    0.257  39.578  -0.347   0.858   0.002   0.139
E N  2KSOA                 80    0.620  90.812   0.774   0.600   0.003   0.397
E T  2KSOA                 81    0.694  96.272   0.737   0.321   0.003   0.675
E V  2KSOA                 82    0.577  88.639  -0.975   0.003   0.003   0.994

Explain the output.  Go back.
```

---
